# Ketoacidosis Risk in Non-diabetic Patients Using Semaglutide Versus Tirzepatide for Obesity: A Disproportionality Analysis of the FDA Adverse Event Reporting System

**DOI:** 10.7759/cureus.108868

**Published:** 2026-05-14

**Authors:** Alisher Makhmutov, Faisal Qureshi

**Affiliations:** 1 Internal Medicine, Ascension Saint Joseph, Chicago, USA; 2 Endocrinology, Diabetes and Metabolism, Ascension Saint Joseph, Chicago, USA

**Keywords:** disproportionality analysis, euglycemic ketoacidosis, faers, glp-1 receptor agonists, obesity, pharmacovigilance, semaglutide, tirzepatide

## Abstract

Background

Glucagon-like peptide-1 receptor agonists, including semaglutide and tirzepatide, are frequently prescribed for obesity management in non-diabetic patients. Euglycemic ketoacidosis has emerged as a concerning complication in this patient population, yet no study has directly compared ketoacidosis reporting signals between these two agents in non-diabetic patients using real-world pharmacovigilance data.

Methods

We conducted a retrospective disproportionality analysis of the FDA Adverse Event Reporting System (FAERS) database from January 2021 through December 2025. Following deduplication, reports were filtered for semaglutide and tirzepatide as primary suspect medications. Non-diabetic patients were identified by excluding reports with diabetes-related indications. Ketoacidosis cases were identified using MedDRA preferred terms. Reporting odds ratios (RORs) and proportional reporting ratios (PRRs) with 95% confidence intervals (CIs) were calculated for each drug.

Results

Following deduplication, 7,349,591 unique reports were analyzed. Among non-diabetic patients, 48,082 semaglutide and 98,295 tirzepatide reports were identified. Ketoacidosis was reported in 261 semaglutide cases and 209 tirzepatide cases. Semaglutide demonstrated a significant disproportionality signal (ROR 3.15, 95% CI 2.78-3.56; PRR 3.14, 95% CI 2.78-3.54). Tirzepatide also showed a significant but lower signal (ROR 1.22, 95% CI 1.06-1.39; PRR 1.21, 95% CI 1.06-1.39). Hospitalization was required in 74.3% and 71.3% of semaglutide and tirzepatide cases, respectively. Tirzepatide-associated reports showed a marked upward trend, increasing from 1-2 cases per quarter in 2022 to 28-34 cases per quarter by 2025.

Conclusions

Both semaglutide and tirzepatide are associated with significant ketoacidosis reporting signals in non-diabetic patients, with semaglutide demonstrating a higher disproportionality signal. The high rates of hospitalization and rapidly increasing tirzepatide reports highlight an emerging safety concern requiring clinical vigilance in patients using these agents for obesity management.

## Introduction

Glucagon-like peptide-1 receptor agonists (GLP-1 RAs) have emerged as therapies in the management of type 2 diabetes and obesity. Among these, semaglutide (Ozempic, Wegovy; Novo Nordisk) and tirzepatide (Mounjaro, Zepbound; Eli Lilly), the latter a dual GLP-1 and glucose-dependent insulinotropic polypeptide (GIP) receptor agonist, have shown strong efficacy in glycemic control and sustained weight loss. With FDA approval of semaglutide for chronic weight management in 2021 and tirzepatide in 2023, their use expanded beyond the diabetic population, with millions of non-diabetic individuals using these agents primarily for obesity management [1-3].

As prescribing has expanded, post-marketing reports have begun revealing safety concerns that were not fully captured in clinical trials. Among these, ketoacidosis, including euglycemic ketoacidosis (EKA), has been increasingly reported in patients using GLP-1 receptor agonists [4-6]. Unlike classic diabetic ketoacidosis, EKA presents with normal or near-normal blood glucose levels, making diagnosis challenging and potentially delaying medical treatment [7]. The proposed mechanisms include GLP-1 RA-induced appetite suppression leading to reduced carbohydrate intake, increased lipolysis, and relative insulin deficiency, all of which promote ketone body accumulation independent of hyperglycemia [8,9].

While ketoacidosis risk has been well characterized with SGLT-2 inhibitors [10], data on GLP-1 RA-associated ketoacidosis specifically in non-diabetic patients remain limited. Existing pharmacovigilance studies have largely focused on diabetic populations or broad adverse event profiles without isolating the non-diabetic subgroup [11,12]. Furthermore, no study to date has directly compared ketoacidosis reporting signals between semaglutide and tirzepatide in non-diabetic patients using real-world pharmacovigilance data.

Antidiabetic medications including GLP-1 receptor agonists and SGLT-2 inhibitors have demonstrated significant cardiometabolic benefits, including improvements in glycemic control, weight reduction, and cardiovascular outcomes [13]. However, the expanding use of these agents across diverse patient populations with varying metabolic profiles has introduced new safety considerations. Metabolic disorders such as insulin resistance, dyslipidemia, and altered ketone metabolism may predispose certain patients to ketoacidotic states when exposed to GLP-1 RAs, particularly in the setting of reduced caloric intake or intercurrent illness. Understanding the interplay between these pharmacologic agents and underlying metabolic vulnerabilities is therefore essential for safe clinical practice.

In this study, we conducted a disproportionality analysis of the FDA Adverse Event Reporting System (FAERS) database from 2021 to 2025 to characterize and compare ketoacidosis reporting signals associated with semaglutide and tirzepatide in non-diabetic patients and to describe their demographic characteristics and clinical outcomes. This work has not been previously presented at any conference or meeting.

## Materials and methods

Study design and data source

We conducted a retrospective pharmacovigilance study using spontaneous adverse event reports from the FAERS database [14]. FAERS is a publicly available spontaneous reporting system maintained by the U.S. Food and Drug Administration that collects voluntary adverse event reports from healthcare professionals, consumers, and manufacturers. The database is organized into quarterly files containing demographic information, drug details, adverse reactions, indications, outcomes, and report sources. Data from January 2021 through December 2025 were included, encompassing 20 quarterly data files. This study period was selected to capture the era of widespread non-diabetic GLP-1 RA prescribing, beginning with the FDA approval of semaglutide for chronic weight management in 2021 and extending through the rapid expansion of tirzepatide use following its approval in 2023.

Data extraction and deduplication

Quarterly ASCII files were downloaded from the FAERS public database and merged across all 20 study quarters using R software version 4.5.3. Each quarterly dataset contains seven relational files: demographic (DEMO), drug (DRUG), reaction (REAC), indication (INDI), outcome (OUTC), report source (RPSR), and therapy (THER). To minimize duplicate reporting bias, deduplication was performed by retaining the most recent report for each unique case identifier (caseid), resulting in 7,349,591 unique reports from an initial 8,598,529 records.

Drug identification

Semaglutide and tirzepatide were identified in the DRUG file using both the drugname and prod_ai fields with case-insensitive string matching. Brand names searched included Ozempic, Wegovy, and Rybelsus for semaglutide, and Mounjaro and Zepbound for tirzepatide. Only reports where the drug was listed as the primary suspect (role_cod = "PS") were included in the primary analysis to ensure that identified adverse events were attributable to the drug of interest rather than concomitant medications.

Inclusion and exclusion criteria

Reports were included if: (1) semaglutide or tirzepatide was listed as the primary suspect drug and (2) the report contained no diabetes-related indications. Reports were excluded if: (1) they were identified as duplicates during deduplication; (2) the drug was listed as a concomitant or interacting medication rather than the primary suspect; or (3) the report contained any diabetes-related indication including type 1 diabetes, type 2 diabetes, T1DM, T2DM, or hyperglycemia in the INDI file.

Outcome definition

Ketoacidosis cases were identified in the REAC file using the following MedDRA preferred terms: "Ketoacidosis," "Diabetic ketoacidosis," "Euglycemic ketoacidosis," "Euglycaemic ketoacidosis," and "Ketosis." These terms were selected to capture the full spectrum of ketoacidosis presentations including euglycemic variants which may not be coded as diabetic ketoacidosis.

Non-diabetic population identification

To isolate non-diabetic patients, the INDI file was queried for diabetes-related indications using the following terms: "diabetes," "diabetic," "type 1," "type 2," "T1DM," "T2DM," and "hyperglycemia." Reports containing any of these indications were excluded from the analysis to ensure the study population represented patients using these medications exclusively for obesity management or other non-diabetic indications. We acknowledge that this keyword-based approach may not capture all patients with diabetes, particularly those with undiagnosed diabetes or incompletely coded indications. To minimize misclassification, we searched for both specific diagnostic terms and general hyperglycemia-related keywords across all indication fields. However, residual misclassification cannot be entirely excluded given the inherent limitations of spontaneous reporting systems.

Statistical analysis

Disproportionality analysis was performed using two established pharmacovigilance metrics: the reporting odds ratio (ROR) and the proportional reporting ratio (PRR), each with 95% confidence intervals (CIs) [15]. The ROR was calculated as (a/b)/(c/d) using a 2×2 contingency table, where a = number of reports for the drug-event combination of interest, b = number of reports for the drug without the event, c = number of reports for the event with other drugs, and d = number of reports for neither the drug nor the event. The PRR was calculated as (a/(a+b))/(c/(c+d)). A safety signal was considered significant when the lower bound of the 95% CI exceeded 1.0. Demographic characteristics including age, sex distribution, and clinical outcomes were summarized using descriptive statistics. Temporal trends in quarterly reporting rates were analyzed to assess changes in reporting patterns over the study period. All statistical analyses were conducted using R software version 4.5.3 (R Foundation for Statistical Computing, Vienna, Austria).

Ethics statement

This study utilized publicly available de-identified data from the FAERS and did not require Institutional Review Board (IRB) approval or informed consent.

## Results

Study population

Following deduplication of the FAERS database (2021-2025), 7,349,591 unique adverse event reports were identified. A total of 72,383 reports were associated with semaglutide and 120,980 with tirzepatide. After excluding patients with diabetes-related indications, 48,082 non-diabetic semaglutide reports and 98,295 non-diabetic tirzepatide reports were identified for analysis.

Ketoacidosis reports

Among non-diabetic patients, 261 ketoacidosis cases were identified in semaglutide users and 209 in tirzepatide users. The total number of ketoacidosis reports across all drugs in the database was 12,549.

Patient demographics

Semaglutide-associated ketoacidosis cases had a mean age of 53.1 years (median 57), with 141 (54%) female and 96 (36.8%) male patients. Tirzepatide-associated cases presented in a younger patient population, with a mean age of 40.6 years (median 36 years), with 134 (64.1%) female and 39 (18.7%) male patients. Demographic and clinical characteristics are summarized in Table 1.

**Table 1:** Demographic and Clinical Characteristics of Ketoacidosis Cases in Non-diabetic Patients

Characteristic	Semaglutide (n=261)	Tirzepatide (n=209)
Mean age, years (median)	53.1 (57)	40.6 (36)
Female, n (%)	141 (54.0%)	134 (64.1%)
Male, n (%)	96 (36.8%)	39 (18.7%)
Unknown sex, n (%)	24 (9.2%)	36 (17.2%)
Hospitalization, n (%)	194 (74.3%)	149 (71.3%)
Life-threatening, n (%)	25 (9.6%)	30 (14.4%)
Death, n (%)	4 (1.5%)	2 (1.0%)
Disability, n (%)	4 (1.5%)	6 (2.9%)
Required intervention, n (%)	3 (1.1%)	4 (1.9%)

Clinical outcomes

Among semaglutide ketoacidosis cases, 194 (74.3%) required hospitalization, 25 (9.6%) were life-threatening, and 4 (1.5%) resulted in death. Among tirzepatide ketoacidosis cases, 149 (71.3%) required hospitalization, 30 (14.4%) were life-threatening, and two cases (1.0%) resulted in death.

Disproportionality analysis

Semaglutide demonstrated a significant ketoacidosis reporting signal with an ROR of 3.15 (95% CI 2.78-3.56) and PRR of 3.14 (95% CI 2.78-3.54). Tirzepatide also showed a statistically significant but notably lower signal, with an ROR of 1.22 (95% CI 1.06-1.39) and PRR of 1.21 (95% CI 1.06-1.39). Full disproportionality analysis results are presented in Table 2.

**Table 2:** Disproportionality Analysis Results ROR: Reporting odds ratio; PRR: proportional reporting ratio

Drug	Total Reports	Non-diabetic Reports	Ketoacidosis Cases	ROR (95% CI)	PRR (95% CI)
Semaglutide	72,383	48,082	261	3.15 (2.78–3.56)	3.14 (2.78–3.54)
Tirzepatide	1,20,980	98,295	209	1.22 (1.06–1.39)	1.21 (1.06–1.39)

Temporal trends

**Figure 1:**
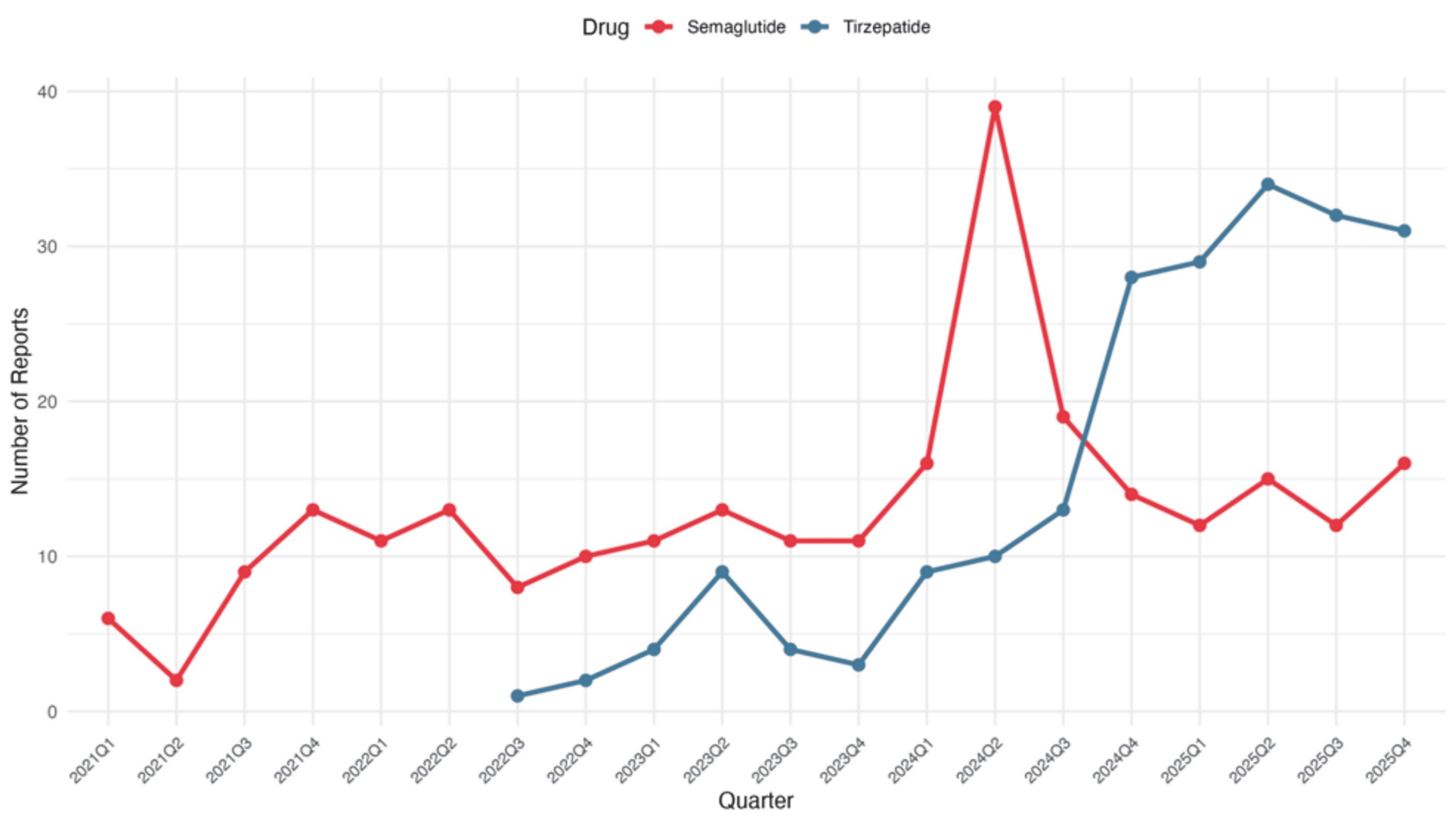
Quarterly Trend of Ketoacidosis Reports in Non-diabetic Patients Using Semaglutide and Tirzepatide, FAERS 2021–2025 FAERS: FDA Adverse Event Reporting System

Quarterly trend analysis revealed distinct reporting patterns between the two drugs. Semaglutide ketoacidosis reports were present throughout the study period with relatively stable quarterly counts. Tirzepatide reports, first appearing in 2022Q3 following FDA approval, demonstrated a marked upward trend, with quarterly cases increasing from 1-2 in 2022 to 28-34 cases per quarter by late 2024 through 2025 (Figure 1).

## Discussion

In this analysis of FAERS data from 2021 to 2025, we found significant ketoacidosis reporting signals for both semaglutide and tirzepatide among non-diabetic patients, with semaglutide showing a stronger signal (ROR 3.15) than tirzepatide (ROR 1.22). To our knowledge, this is the first study to directly compare ketoacidosis reporting signals between these two agents, specifically in the non-diabetic population using real-world pharmacovigilance data.

These findings carry real clinical weight given how rapidly GLP-1 RAs have expanded into the non-diabetic obesity market. Both semaglutide (Wegovy) and tirzepatide (Zepbound) have received FDA approval for chronic weight management in patients without diabetes [1,2], and their use in this population has grown substantially in recent years [3]. Unlike diabetic patients, non-diabetic users may be less likely to monitor blood glucose, potentially delaying recognition of euglycemic ketoacidosis, where glucose levels may be normal or only mildly elevated [7].

The proposed mechanism for GLP-1 RA-associated EKA involves several interconnected pathways. GLP-1 RAs suppress appetite and reduce caloric intake, potentially inducing a starvation-like state that promotes lipolysis and ketogenesis [8,9]. This is further compounded by the nausea and vomiting that many patients experience on these medications, which can dramatically reduce carbohydrate intake and accelerate ketone accumulation [6]. Unlike SGLT-2 inhibitor-associated EKA, which is driven primarily by glucosuria and volume depletion [10], GLP-1 RA-associated EKA appears to be predominantly driven by caloric restriction and relative insulin deficiency.

The notably higher ROR for semaglutide compared to tirzepatide (3.15 vs 1.22) is an interesting finding that warrants further investigation. Several explanations may account for this difference. First, semaglutide has been available longer in the non-diabetic population, potentially leading to higher absolute reporting rates. Second, semaglutide causes more pronounced gastrointestinal side effects compared to tirzepatide [16], which may contribute to greater caloric restriction and dehydration, increasing ketoacidosis risk. Third, the dual GIP/GLP-1 mechanism of tirzepatide may modulate glucagon secretion differently than pure GLP-1 agonism, potentially offering some protective effect against ketogenesis, though this remains speculative. It is important to note that disproportionality signals such as ROR reflect reporting patterns rather than true clinical incidence rates. Given the inherent limitations of spontaneous reporting systems, including reporting bias and absence of denominator exposure data, the higher ROR observed with semaglutide should not be interpreted as definitive evidence of greater clinical risk compared to tirzepatide. These findings are hypothesis-generating and require confirmation through prospective studies with controlled exposure data.

Despite tirzepatide's lower relative risk signal, the trend data tell a different story; tirzepatide-associated ketoacidosis reports have increased dramatically since 2022, reaching 28-34 cases per quarter by late 2024 through 2025, compared to 1-2 cases per quarter at its market introduction. This trajectory suggests that as tirzepatide prescriptions continue to grow, the absolute burden of ketoacidosis cases will likely increase substantially, even if the relative risk remains lower than semaglutide.

The demographic profile of affected patients provides additional clinical context. Semaglutide-associated cases were older (mean age 53.1 years) while tirzepatide cases were notably younger (mean age 40.6 years), potentially reflecting differences in the prescribing patterns and patient populations for each drug. The predominance of female patients in both groups, 141 (54%) for semaglutide and 134 (64%) for tirzepatide, is consistent with the higher rates of obesity treatment-seeking behavior among women [3]. The high rates of serious outcomes, including hospitalization in over 70% of cases and life-threatening events in 9.6-14.4%, underscore the clinical severity of this adverse event.

Several limitations of this study merit consideration. FAERS is a spontaneous reporting system subject to underreporting bias, meaning the true incidence of ketoacidosis is likely higher than captured in our analysis. Causality cannot be established from pharmacovigilance data alone. The identification of non-diabetic patients relied on keyword filtering of indication fields in FAERS, which may not capture patients with undiagnosed diabetes or incompletely documented indications, potentially resulting in residual misclassification of some diabetic patients within the non-diabetic cohort. Confounding factors could not be controlled for in this analysis; in particular, concomitant SGLT-2 inhibitor use, which independently increases ketoacidosis risk, could not be excluded from our non-diabetic cohort, and dietary factors such as low-carbohydrate or ketogenic diets, which are common among patients using GLP-1 RAs for weight loss, may have contributed to ketoacidosis risk independently of drug exposure. Finally, differences in market exposure time and prescription volume between semaglutide and tirzepatide may partially explain the observed differences in reporting signals rather than true pharmacological differences.

## Conclusions

Both semaglutide and tirzepatide demonstrated significant ketoacidosis reporting signals in non-diabetic patients in the FAERS database, with semaglutide showing a substantially higher disproportionality signal than tirzepatide. The majority of cases resulted in hospitalization or life-threatening outcomes, highlighting the clinical severity of this adverse event. Given the rapidly increasing use of these agents for obesity management in non-diabetic populations, clinicians should maintain awareness of ketoacidosis as a potential complication, particularly in patients with reduced oral intake, active vomiting, or those following low-carbohydrate diets. Prospective studies are needed to establish causality, identify high-risk patients, and guide clinical monitoring protocols.
